# Classification and evaluation of educational apps for early childhood: Security matters

**DOI:** 10.1007/s10639-022-11289-w

**Published:** 2022-08-24

**Authors:** Julie Vaiopoulou, Stamatios Papadakis, Eirini Sifaki, Michail Kalogiannakis, Dimitrios Stamovlasis

**Affiliations:** 1 Faculty of Education, Department of Preschool Education, University of Crete, Rethymno, Greece; 2 Department of Education, University of Nicosia, Nicosia, Cyprus; 3 School of Humanities and Social Sciences, Department of Language & Intercultural Studies, University of Thessaly, Volos, Greece; 4 Department of Philosophy and Education, Aristotle University of Thessaloniki, Thessaloniki, Greece

**Keywords:** Educational apps, Classification, Latent class analysis, Evaluation, Higher education, Security

## Abstract

This study explored certain popular educational apps’ vital characteristics and potential profiles (*n1 =* 50) for kindergarten kids. The profile analysis involved a categorization ascended from an evaluation process conducted by pre-service early childhood teachers’ (*n2 =* 295) at the University of Crete, Greece, using a new instrument, validated in the present research, the ETEA-2 scale. The categorization criteria were the five dimensions of the ETEA-2: Learning, Suitability, Usability, Security, and Parental Control. The classification based on Latent Class Analysis led to three apps' profiles: Cluster/profile 1 includes apps that have high values in Learning, Usability, Suitability, and medium Parental Control and Security; Cluster/Profile 2 includes apps with medium Learning, Usability, Suitability, but low Parental Control and High Security; Cluster/Profile 3 includes apps with medium Learning, Usability, Suitability, but low Parental Control and low Security. This profile scheme is an indicative categorization summarizing the crucial features that popular apps possess and can help parents and/or educators’ decision-making on choosing the desirable application for their kids. Moreover, from an independent evaluation of these specific fifty apps sought on the internet, the members of Cluster2/Profile 2 were the most popular and preferable, as suggested by the number of downloads. This profile is distinguished for the security dimension.

## Introduction

In the last decade, smart mobile technology has become popular among young kids (Nikolopoulou, 2020), as over 50% of the available educational apps target preschool children (Callaghan & Reich, 2018). Historically, this trend was combined with an expressed need for excellence in teaching, promoting digital technologies addressed to young children, and a new educational orientation supported by politicians (Bourbour, 2020). Early childhood education does not ignore interactive smart screen technologies, and a special interest in researching how these devices can improve the learning procedure in formal and informal settings has emerged (Tavernier, 2016). A growing body of research into preschool children’s smart tools states that digital devices have become pervasive, whilst educators respond pedagogically to the new opportunities, constantly incorporating these types of devices into their daily teaching practice (Fleer, 2020).

Preschoolers tend to express few issues in navigating touchscreen devices, so they feel motivated to use them in diverse ways, such as engaging in reading digital books, even if they were not interested in reading print books. Many educators seek novel ways to motivate learning by incorporating interactive mobile technology in their classes (Eutsler & Trotter, 2020). Applications (apps) can support children’s active engagement by embedding educational concepts into game-like activities. They can also scaffold children’s learning through adaptive learning technology, provide feedback and rewards through gameplay, and promote a repeated practice of critical foundational skills (Griffith & Arnold, 2019; Hirsh-Pasek et al., 2015b).

However, the quantity expansion has not seen an associated increase in quality. Studies on the usability of tablet games for children typically occur in laboratory settings, and they do not understand the context of use (Read et al., 2018). Designing technology for children comes with unique ethical challenges and responsibilities related to the inclusion of children in the research and design processes and the outcomes of that work (Frauenberger et al., 2018). Unfortunately, most apps that parents and teachers find under the “educational” category in app stores have no evidence of efficacy. Instead, they primarily target rote academic skills, use little or no input from developmental specialists or educators, and are rarely based on established curricula (Guernsey & Levine, 2015). Most of these apps treat children as young adults, not considering the unique needs of this age group, such as the fine motor skills needed to handle an app. This is particularly important as children constantly engage with technology, which usually lacks quality in aligning with their developmental level or needs (Callaghan & Reich, 2018; Plowman et al., 2012).

Educators need support in the choice of apps with educational values. Choosing an app can be overwhelming; the educational category in app stores often does not help educators find developmentally appropriate apps. In examining the educational value, many of the apps tagged appropriately for young children were inadequate and often mislabeled the acceptable age range (Fantozzi, 2021; Sari et al., 2017, 2019). Apps are a new pedagogical tool, and, as such, they should be subjected to the same “quality control” to ensure their effectiveness in teaching kids in a developmentally appropriate way. Those concerns led the researchers, during the past years, to investigate further the process of designing apps aiming at children’s engagement in learning (Callaghan & Reich, 2018).

## Literature review

The recent development of many latest information and communication technologies has significantly changed these technologies’ role in daily life (Nguyen et al., 2014). Indeed, statistics show that about 75% of the kids in the USA, as young as four years old, own a personal smart mobile device (Kabali et al., 2015). Following this trend, children in the UK favour touchscreen devices over desktop computers or even laptops (Ofcom, 2020). Studies have shown that, by the age of three years old, children can tap and swipe independently on such devices (Marsh et al., 2015; Vatavu et al., 2015), an ability that can support them to learn independently, either in traditional or informal learning environments (Booton et al., 2021).

New mobile technologies have propelled apps and software supporting m-learning (Luna-Nevarez & McGovern, 2018). When Apple’s iTunes App Store and Google’s Android Market first launched in 2008, smartphone users could choose from about 600 apps (Federal Trade Commission, 2012). More than 470 million educational apps are now in the Apple App store and 466 million in the Android Market. During the first quarter of 2020, the COVID-19 pandemic caused a surge in downloads of educational apps (Leci, 2021). With the advancement of interactive technology and more user-friendly touchable interfaces, these devices in early-year classrooms appropriately prepare young children for the 21st century (Miller, 2018). The new digital mobile technologies bear some advantages for incorporating them in educational settings; they are portable. Their touchscreens are easy to use and require a small workspace while allowing for multiple people viewing (Lawrence, 2017). Compared to mouse-operated computers, a tablet’s accessible touch-based operational features (e.g., tap, slide, swipe) make it intuitive. They are convenient due to their portability and size. The attractive multimodal features of apps (e.g., animations, audio, colourful graphics, highlighted texts) stimulate a child’s visual, auditory, kinesthetic, and tactile senses and deliver immediate feedback. The interactive nature of tablets gives children the autonomy and agency to select their activities. The multi-functionality of tablets increases the chance of capturing a child’s interest, offering a range of activities with one device (Neumann, 2020). Thus, these apps are often called ‘edutainment’ due to their formats that resemble games, the visual aids they provide, and their didactic approach to teaching educational content (Okan, 2003).

Most children use interactive smart screen technologies to achieve different purposes, some of which may be described as playful, cooperative, and interactive (Marsh et al., 2018). The Joan Ganz Cooney Center named five ways mobile media devices and apps can change children’s education. They range from the ‘anywhere, anytime’ type of ubiquitous learning (i.e., quick and ready access to technologies). Thus, it promotes situated learning and breaks the barrier between home, school, and after school. It can fit with diverse environments to improve 21st-century social interactions by enabling a personalized experience to reach underserved children (Looi et al., 2011; Shuler, 2009).

Among the scopes of preschool education is the development of children’s early literacy and numeracy skills. These skills are known predictors of later academic achievement and advance through developmentally appropriate apps (Hoareau et al., 2021). These apps focus on the excellent design of open-ended and child-driven activities to engage young students in learning (Hirsh-Pasek et al., 2015a; Reed & Takeuchi, 2011). The appropriateness of the developmental aspect of the software used significantly impacts children’s education because developmentally inappropriate software can negatively affect children’s creative skills. For instance, interfaces for young children should be highly visual, avoiding text as much as possible to reduce cognitive load (Soni et al., 2019). Appropriate apps should include prosocial content, non-violent stories, and characters, promote diversity in terms of gender and culture, and have low levels of advertising. Content needs to have the right place for young children to enhance their executive function instead of hindering it (Papadakis, 2021). Given suitable apps, educators can consider how different apps meet children’s play interests. Also, they can create new opportunities for children to mix and match modes of communication in digital forms such as video, audio, images, and text, which is vital for sustaining children’s in-app digital play (Troseth et al., 2016).

The rapid changes in communication forms have created an environment where parents and educators of young children find themselves in an unknown context, which heavily demands new everyday practices. They must familiarize themselves with the new trends, find easily accessible mobile technologies and support ways to make them attractive and available to young children (Laidlaw et al., 2019). To provide children with high-quality apps that promote play and creativity, caregivers should be able to assess the quality of apps so that they can buy the best in terms of suitability and educational value (Marsh et al., 2018). There are about 80,000 apps promoted as ‘educational’ (Healthy Children, 2018). However, researchers agree that most children’s apps advertised lack educational value and any foundation in relevant studies outcomes (Ólafsson et al., 2013). Indeed, educational benefits are rarely known as few edutainment games are assessed for their benefits or outcomes (Guernsey & Levine, 2015; Hirsh-Pasek et al., 2015b; Nikolayev et al., 2020).

Furthermore, due to design limitations, e.g., poor interface design practices and lack of guidance and feedback during the interaction, children might have difficulty learning the content as intended (Soni et al., 2019). Another crucial factor is that, even though the Android Play Store, over 95% of apps were classified as ‘free’, the cost of developing and marketing mobile apps is substantial, so, usually, alternative forms of revenue generation (monetization) are often necessary for developers in this context. Most mobile apps can be used free of charge, thus including alternative ways of monetization, such as advertising (Fitton & Read, 2019). The implications of in-app advertisements and buying opportunities for adults have been studied. However, despite the well-documented susceptibility of younger users to manipulation, they have received comparatively little consideration (Fitton & Read, 2019).

The above defaults pose severe challenges for educators and parents in finding high-quality apps (Livingstone et al., 2018). Thus, caregivers could be assisted from a tool designed to evaluate educational apps based on early years learning theory. Such an instrument could also aid app developers in ensuring that their products consist of high-quality elements (Kolak et al., 2021). In this context, the present research investigates the features of effective educational apps. It provides classification and evaluation of several prevalent cases, revealing the crucial criteria that guide parent and/or educators’ selection.

## The present research

### Aim and research questions

The aim of the present endeavour is three-fold. The first aim is to present an evaluation instrument for apps and their validity and reliability accounts. The next goal is to achieve a valid classification of several existing popular app profiles, that is, to reveal potential distinct groups with specific characteristics measured by the dimensions of the above-proposed instrument. Note that such classification is not a priori valid, and the hypothesized existing clusters/profiles should be verified by a proper method. Latent Class Analysis (LCA), a statistical model-based method, was used in this study thus, the findings can be generalized as prevailing tendencies in the population. The potential profiles/trends will reflect the existing apps’ aggregated features by design. While a third aim is to investigate which of the ensuing profiles is the most popular or preferable among parents. Based on the above aims, the following research questions were posited:

- RQ1: Does the proposed instrument for evaluating apps provide valid and reliable measures?
- RQ2: Based on the dimensions of the proposed instrument, can a valid classification of the selected apps be derived?
- RQ3: What are the characteristics of the ensuing profiles (if any)?
- RQ4: Is there any apps profile more prevailing in terms of popularity among caregivers? 

### Procedures and data collection

The present study followed international research ethics guidelines (Petousi & Sifaki, 2020).

#### Selection of apps

For the selection and apps analysis, the procedure described previously in the literature was adapted (Soni et al., 2019), leading to a two-phase process. From the ‘Children’ category apps that belonged to Education or Games in the Google Play app store 200 free apps were randomly selected between February and May 2021. In the next step, the following exclusion criteria were used to find the apps targeted at the intended audience:

- Apps with fewer than 500 user ratings were removed.
- Apps intended to be co-used with an adult were removed.
- Apps targeted at children with disabilities were excluded.

The final dataset included 50 apps spanned a mix of games (56%) and educational (44%) apps (see Tables 6 and 7 in Appendix 1). Educational apps included storytelling or teaching children how to write the alphabet.

#### Apps evaluation phase - data collection

Pre-service early childhood teachers evaluated the fifty selected apps for an educational technologies course. The participants were students (*n* = 295, 99% females; aged 21–22) at the University of Crete, Department of Preschool Education, Greece, who were familiar with the educational apps and they downloaded the 50 educational apps for preschool-aged children to their smart portable devices. The research followed the University of Crete’s ethical practice guidelines and was approved by the respective Institutional Review Board.

The participants had to fill out a specific evaluation questionnaire (five-point Likert scale), which included selected items and dimensions of two validated instruments developed by the authors: the scale *Evaluation Tool for Educational Apps* [ETEA] (Papadakis et al., 2020) and the scale *Perceptions about Educational Apps Use–parents* [PEAU-p] (Vaiopoulou et al., 2021). The instrument implemented in this research endeavour is ETEA-2, and its factorial validity is verified with the empirical data.

#### Data analysis

The Instrument: ETEA-2 comprises of 22 items organized into the structure of five dimensions, i.e., Learning, Suitability, Usability, Security, and Parental Control. Principal Components Analysis (PCA) was applied to the present collected dataset reproduced the fundamental five-dimensional structure. Bartlett’s test of sphericity and KMO criteria suggested adequate variance for applying factor analysis [*χ*^*2*^ = 14730.587, *d.f. =*201, *p <* .001; *KMO* = 0.908]. The Kaiser criterion (eigenvalue > 1) was used for selecting the number of factors, and only items with factor loadings greater than 0.50 were kept. The same factor structure is obtained by applying the principal axis factoring method, whereas varimax rotation was used. Table 1 depicts the five-factor structure, which explains 64.42% of the total variance. The corresponding eigenvalues for Learning, Suitability, Usability, Security and Parental Control are 4.91, 3.49, 2.20, 1.45, and 1.43, respectively, while the corresponding percentages of the explained variance are 23.38, 16.64, 10.46, 7.36, and 6.81, correspondigly. Table 1 also shows Cronbach’s alpha coefficients for Learning, Suitability, Usability, Security, and Parental Control (0.903, 0.855, 0.711, 0.668, and 0.674, respectively).

**Table 1:** Structure Factors of ETEA-2. Results from PCA with varimax rotation

	Dimensions				
Items	Learning	Suitability	Usability	Security	Parental control
17. Contributes to cognitive development.	0.859				
14. Facilitates new knowledge acquisition.	0.842				
21. It is complementary to traditional teaching.	0.812				
22. Creates a complete learning environment.	0.790				
12. Promotes logical thinking.	0.701				
15. Enhances language development.	0.697				
16. Promotes creative thinking.	0.673				
19. Includes pleasant sounds for kids.		0.774			
10. Includes pleasant pictures for kids.		0.738			
8. Provides feedback easily comprehensible for kids.		0.679			
7. Offers many ways of presenting information (sounds, pictures, talks).		0.651			
6. Provides multimedia choices suitable for kids.		0.612			
1. Guidelines are comprehensible for kids.		0.584			
2. The visual elements are presented consistently throughout the application.			0.796		
3. The app elements (menu, buttons, and arrows) are correctly placed and facilitate use by kids.			0.704		
4. The kids can use the app without adults’ guidance.			0.588		
11. Urges the child to proceed with online financial transactions (of any kind).				0.847	
18. Contains elements interrupting the flow (e.g., pop-up messages, advertisements).				0.831	
9. Provides advice to parents.					0.791
20. Informs parents about the child’s progress (e.g., via e-mail).					0.627
13. The app explicitly states to parents the personal data management policy.					0.571
Cronbach’s alpha	0.903	0.855	0.711	0.668	0.674

Table 2 shows the correlation matrix for the five components and their means and standard deviations. Descriptive statistics on the evaluation scores of each app in the five dimensions are presented in the Appendix 1. The fifty apps under investigation can be ordered according to their scores and evaluated in each dimension. However, a classification scheme that considers the five dimensions factors is more effective. Thus, in the next step, the five dimensions were used as the basis for cluster analysis to classify the apps. The data were processed using Latent Class Analysis, a model-based cluster analysis that allows the resultant groups to be generalized as existing trends in the population (Magidson & Vermunt, 2004; Stamovlasis et al., 2018).

**Table 2:** Correlation matrix and descriptive statistics for the five dimensions

Variable	Learning	Usability	Suitability	Parental control	Security
1. Learning	1				
2. Usability	0.28***	1			
3. Suitability	0.64***	0.51***	1		
4. Parental Control	-0.006	0.17***	0.08**	1	
5. Security	-0.12**	0.02	-0.07*	0.051	1
Mean	3.029	3.762	3.582	2.023	2.806
SD	0.921	0.759	0.888	0.872	1.383

*p < .05, **p < .01, ***p < .001

#### Latent class analysis

Latent Class Analysis (LCA) was applied to classify the fifty apps into distinct groups/clusters sharing typical profiles based on characteristics measured via the implemented evaluation instrument. Specifically, the averages of 295 evaluation scores in the five dimensions were used (in the Appendix 1). LCA provides several indicators for assessing the model-goodness-of-fit: i.e., the number of parameters, likelihood ratio statistic (L2), Bayesian Information Criterion (BIC), Akaike’s Information Criterion (AIC), degrees of freedom, and classification error (Magidson & Vermunt, 2004). Table 3 shows the statistical indexes for one-, two-, three-, four- five- and six-cluster solutions. The three-cluster model is the best parsimonious since it shows the lowest classification error and the lowest Bayesian Information criterion-BIC value.

**Table 3:** Results of the LCA

	LL	BIC(LL.)	AIC (LL.)	Npar	Class.Err.	
1-Cluster	-166.12	371.35	352.23	10	0	
2-Cluster	-132.49	347.12	306.97	21	0.0310	
3-Cluster*	-108.35	341.89	280.71	32	0.0282	
4-Cluster	-90.73	349.67	267.45	43	0.0324	
5-Cluster	-77.19	365.63	262.38	54	0.0203	
6-Cluster	-63.99	382.27	257.98	65	0.0236	

**Fig. 1:**
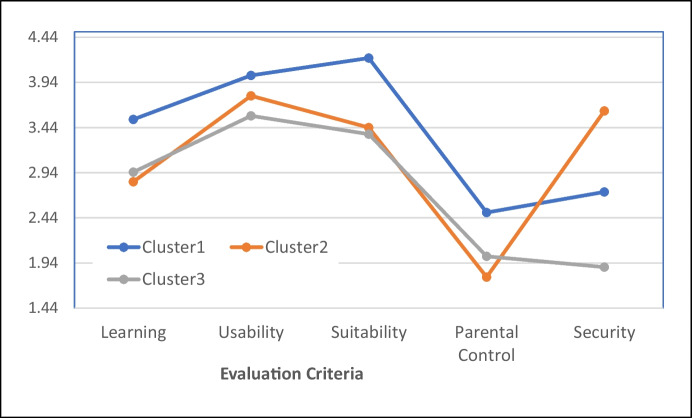
The three clusters/profiles are based on the five dimensions of ETEA-2

Figure 1 presents the ensued profiles based on the dimensions of the instrument. Cluster/Profile 1 (size = 11) includes apps with high Learning, Usability, Suitability, and medium Parental Control and Security values. Cluster/Profile 2 (size = 21) includes apps with medium Learning, Usability, Suitability, low Parental Control, but high Security. Cluster/Profile 3 (size = 18) includes apps with medium Learning, Usability, and Suitability but low Parental Control and Security. Table 4 shows a qualitative description of the resulting clusters/ profiles on their values in the five dimensions where high, medium and low denote relative levels.

**Table 4:** A qualitative description of the ensued clusters/profiles

	Cluster1	Cluster2	Cluster3
Cluster Size	11	21	18
Dimensions			
Learning	High	Medium	Medium
Usability	High	Medium	Medium
Suitability	High	Medium	Medium
Parental Control	Medium	Low	Low
Security	Medium	High	Low

**Fig. 2:**
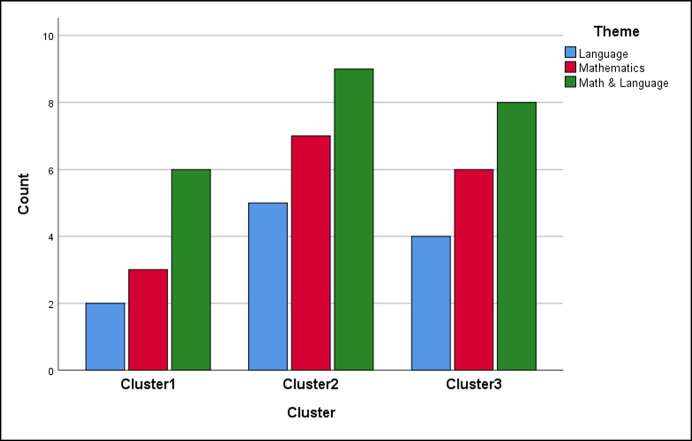
The distributions of the varying theme of apps within each cluster/profile

Table 5 shows the allocation of the specific fifty apps used (ID numbers, 1 to 50) into the three cluster profiles. The theme content of the app concerned Math, Language, or Both. The distributions of the varying themes within each cluster/profile are analogous, as shown in Fig. 2.

**Table 5:** Classification of the 50 apps into the three clusters/profiles and their mean values in each of the five dimensions

	Cluster 1	Cluster2	Cluster3
Cluster Size	11	21	18
App ID	2, 9, 10, 12, 13, 17, 18, 19, 33, 46, 48	1, 3, 4, 5, 6, 7, 8, 14, 20, 25, 26, 28, 34, 35, 36,37, 41, 42, 43, 47, 49	11, 15, 16, 21, 22, 23, 24, 27, 29, 30, 31, 32, 38, 39, 40, 44, 45, 50
Dimensions		Mean values	
Learning	3,53	2,84	2,95
Usability	4,02	3,79	3,57
Suitability	4,21	3,44	3,37
Parental Control	2,50	1,78	2,01
Security	2,73	3,62	1,89

**Fig. 3:**
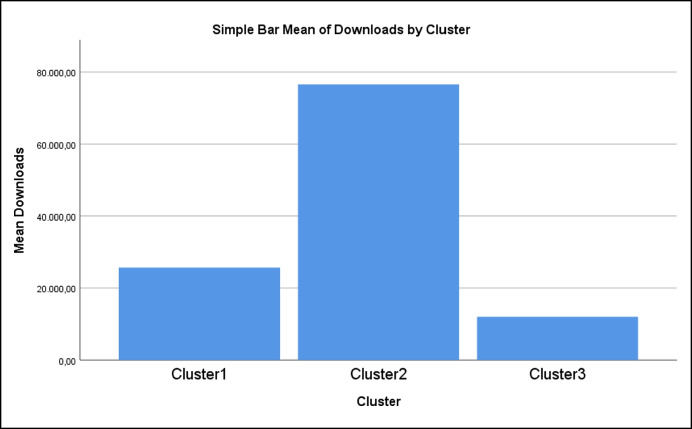
The mean number of downloads for the three clusters/profiles shows the superiority of Cluster / Profile 2, which emphasizes the Security dimension

Moreover, an independent evaluation of these specific fifty apps on the internet was sought, based on scores indicating the most popular (in terms of ratings) and the most downloadable (in terms of total downloads as provided by the platforms) apps. Interestingly, the members of Cluster2, which belong to the profile that is distinguished for scoring higher in the security dimension, are by far the most popular and the most downloadable apps (Fig. 3). This finding is of paramount importance for anyone involved in the application of mobile technology for young children in informal or formal educational environments and the developers and designers of apps that are informed about the crucial demand set by parents and teachers.

## Discussion

Concluding on the research question (RQ1-RQ4), it is clear from factor analysis and internal consistency measures that the proposed instrument conforms to validity and reliability requirements (RQ1). By implementing Latent Class Analysis based on the five dimensions of the proposed questionnaire, a valid classification of the selected apps was derived (RQ2). This led to three emergent profiles, which are understood within the dimensionality of the evaluation system (RQ3). Auxiliary analysis showed that profile 2, which scores higher in security issues, appears as the most popular or desirable among parents or teachers (RQ4). The findings of the present research contributes to the relevant literature and enrich the ongoing discussion on app use and its incorporation into the formal or informal educational process.

Smart interactive technologies are already recognized for improving the quality of the learning process mediated by devices and wireless networked technologies (Pondee et al., 2021). The mobile apps marketplace constantly evolves with new media forms claiming to equip educators and parents with tools for their children and students’ entertainment and education. The two major app stores provide app information and controls, but this is not enough. All app ecosystem parts – the app stores, the developers, and the third parties providing services within the apps – must do more to ensure that educators and parents have access to clear, concise, and timely information about the available apps (Federal Trade Commission, 2012). Given that children can learn from well-designed educational apps, this signals a new challenge for developers to create apps with quality content. In this sense, the quality of tablet app experiences, when it facilitates developmentally appropriate learning practices, may be more important than the time spent using tablets. Thus, rather than focusing merely on screen time statistics, a deeper and more differentiated analysis is required. Researchers should focus on analyzing child device usage and how this usage is associated with literacy and language learning outcomes (Neumann, 2020).

The present study results align with the findings presented earlier in the relevant literature, despite the differences in the games, targeted ages of the players, media platforms, sample sizes, and countries of origin. Specifically, simple feedback types, such as verification, seemed to be the most popular feedback in other digital media for children, as Blair (2013) and Callaghan and Reich (2018) found. The same studies also noted the small number of games that offered some facilitating or scaffolding. The discrepancies between negative and positive feedback were similar to the findings of Benton and colleagues (Benton et al., 2018). The lack of positive feedback for motivation and encouragement was consistent with Fong et al.‘s (2019) study. These findings highlight that content designers’ potential reliance on standard conventions or design strategies that might not be most beneficial for children’s learning (Nikolayev et al., 2020).

## Concluding remarks: Security matters

Teachers play a decisive role in technology success in schools, and their experiences in teacher education programs influence how they subsequently use technology (McGarr & Gallchóir, 2020). Nevertheless, mobile games to support teaching and learning pedagogies still challenge teachers. Thus, pre-service teachers require specific knowledge to design meaningful learning experiences with mobile games and pedagogically implement them in their teaching (Pondee et al., 2021).

Furthermore, updated guidelines or checklists that teachers can evaluate apps are essential. Such approaches should call for apps to be age-appropriate, have clear instructions, hold well-designed multimedia and interactive features, and intuitive operational features for young children. They must also warn against violent characters or actions, negative social values, and gender stereotyping (Gromik & Litz, 2021). Teachers should check literacy and language apps used in the classroom against interactivity, usability, cultural awareness, collaboration, language and literacy content, and learning outcomes (Neumann, 2020). The present work contributes to evaluation issues by presenting ETEA-2, a valid five-dimensional instrument that forms the proposed criteria for assessing educational apps. These criteria, the proposed dimensions, originate from scrutinizing and elaborating on the perceived challenges and advantages of apps in the literature i.e., the factors influencing the attitudes and preferences of the users. The evaluation process was carried out by teacher-students, who, even though are not experts, will most likely be the final recipients in educational settings.

In addition, by applying LCA, a model-based cluster analysis calculates fifty popular apps into three distinct groups/ profiles, being trends that share specific characteristics by design. Profile 1, having at least medium security and parental control, is rated higher in learning, usability, and suitability. Profile 3 has medium learning, usability and suitability, low security and parental control. Profile 2 differs from Profile 3 in displaying high security characteristics. Profile 1 appears to have the highest overall value. However, it is not the most popular or desirable one. Profile 2 takes this position.

Moreover, on behalf of early childhood teacher educators, efforts should be made to promote the use and applicability of technology in a responsible and developmentally proper way (Nikolopoulou, 2020), by introducing the students to evaluation instruments and educating them to use the effectively. In this digital pedagogical enterprise, it is imperative to endorse all the three protagonist components involved: the technology itself, evaluated according to the educational or entertainment goals; the child to whom it is addressed; and the omnipresent adults (teachers, parents, or caregivers) who guide learning and demand their share in control and safety issues.

Overall, this paper flags the need for a systematic evaluation of mobile apps adressed to young children. Although the looked-for learning outcomes, usability and suitability issues are already acknowledged, the findings signify that security matters more to caregivers and convey the message to the present and future app creators to adopt a more rigorous approach in designing educational apps, giving special consideration to safety.

### Limitations

Although the current research study relied on rigorous statistical methods, it is not without limitations, arising from specific choices and peculiarities of the procedures. First, the pre-service teachers, who served as evaluators, comprised a convenience sample that happened to be already educated and partly informed in educational technology. The vast majority were females; thus, this study can explore no gender differences. In addition, the findings could not be generalized to other countries, cultural backgrounds, and, of course, both genders. Finally, the apps were selected randomly. This limitation, albeit important, is mitigated by the numerous  studies around the globe highlighting the low quality of the self-proclaimed educational apps.

## Data Availability

The datasets generated during and/or analysed during the current study are available from the corresponding author on reasonable request. The authors declare that all data supporting the findings of this study are available within the article and its supplementary information file (Appendix 1).

## Appendix 1

**Table 6:** Descriptive statistics for the evaluation scores of each app in the five dimensions

	Learning		Usability		Suitability		Parental control		Security	
APP id	Mean	SD	Mean	SD	Mean	SD	Mean	SD	Mean	SD
1	3.443	0.682	4.147	0.541	4.287	0.408	1.598	0.737	4.103	0.724
2	3.652	0.742	3.883	0.604	4.278	0.441	2.244	1.072	4.100	0.932
3	2.641	0.713	3.766	0.774	3.355	0.779	1.806	0.792	3.935	1.093
4	3.025	0.719	3.672	0.575	3.230	0.932	1.747	0.705	3.655	1.070
5	3.332	0.724	3.982	0.585	3.821	0.659	1.714	0.633	3.589	1.081
6	3.310	0.803	4.190	0.498	4.172	0.539	1.782	0.686	3.741	1.123
7	2.606	0.707	3.534	0.706	2.983	0.816	1.759	0.712	3.086	1.233
8	3.345	0.701	3.716	0.671	3.759	0.794	1.862	0.848	3.534	1.187
9	3.621	0.691	4.078	0.571	4.218	0.606	2.161	1.006	2.397	1.003
10	4.069	0.710	4.224	0.532	4.586	0.505	2.552	0.948	2.638	1.051
11	2.867	0.705	3.836	0.669	3.793	0.812	1.667	0.729	1.328	0.539
12	3.493	0.613	4.267	0.601	4.356	0.602	2.540	1.103	2.586	1.053
13	3.350	0.680	3.983	0.578	4.063	0.585	2.828	1.274	3.224	1.373
14	2.759	0.762	4.017	0.767	3.529	0.718	1.747	0.800	3.586	1.408
15	3.478	0.859	3.690	0.798	4.149	0.736	1.724	0.685	1.862	0.833
16	3.138	0.858	3.888	0.618	3.810	0.734	1.586	0.688	1.983	0.818
17	3.650	0.892	4.078	0.555	4.224	0.596	2.724	1.047	3.207	1.373
18	3.133	0.931	3.966	0.581	3.822	0.745	2.230	1.124	3.207	1.353
19	3.394	0.895	4.147	0.636	4.080	0.629	2.034	0.726	1.948	0.806
20	3.030	0.866	3.741	0.769	3.707	0.634	1.759	0.672	2.914	1.254
21	2.901	0.864	3.043	0.641	3.040	0.898	1.920	0.694	2.483	1.169
22	2.759	0.775	2.974	0.808	3.017	0.821	1.701	0.651	1.466	0.654
23	3.000	0.927	3.397	0.803	3.586	0.814	2.149	0.962	2.345	1.010
24	3.172	0.869	3.483	0.842	3.672	0.758	1.977	0.895	2.190	0.761
25	3.148	0.933	3.629	0.737	3.649	0.787	1.885	0.632	3.948	1.072
26	2.365	1.052	3.569	1.028	2.879	0.757	1.598	0.651	3.552	0.985
27	3.327	0.632	4.018	0.745	3.435	0.818	1.952	0.963	2.107	0.936
28	3.010	0.785	3.608	0.883	3.150	0.677	2.122	0.937	3.833	0.932
29	2.404	0.564	4.000	0.612	3.466	0.646	1.747	0.716	1.293	0.509
30	3.034	0.894	3.802	0.757	3.316	0.816	1.701	0.632	1.914	0.757
31	2.980	1.073	3.276	0.822	2.161	0.739	2.287	0.958	1.069	0.371
32	2.990	1.084	3.259	0.846	2.207	0.793	2.356	1.123	1.017	0.093
33	4.030	0.632	4.009	0.656	4.569	0.444	2.724	1.212	1.000	0.000
34	2.552	0.748	3.750	0.779	3.155	0.706	1.805	0.588	3.621	1.075
35	2.936	0.933	3.362	0.721	3.833	0.877	1.736	0.663	3.603	1.339
36	2.675	0.800	3.698	0.760	3.351	0.735	1.678	0.560	3.948	1.113
37	2.177	0.759	3.922	0.720	2.862	0.694	2.034	0.499	3.448	1.298
38	2.704	0.873	3.991	0.721	3.270	0.831	2.057	0.564	2.224	1.131
39	2.655	0.851	3.836	0.602	3.626	0.791	2.368	0.906	2.034	0.844
40	3.222	0.878	3.448	0.689	3.672	0.848	2.138	0.538	1.983	1.004
41	2.773	0.910	4.000	0.620	3.374	0.757	1.828	0.670	2.862	1.281
42	2.507	0.814	3.897	0.660	2.948	0.788	1.724	0.598	3.983	1.184
43	2.916	0.885	4.138	0.596	3.477	0.711	1.747	0.621	3.810	1.145
44	2.621	0.818	3.681	0.641	3.448	0.679	2.517	0.889	1.707	0.750
45	3.084	0.840	3.672	0.678	3.661	0.620	2.046	0.582	2.310	1.004
46	3.325	0.932	3.845	0.715	4.149	0.720	2.782	0.870	2.862	1.356
47	1.828	0.670	3.888	0.680	3.069	0.726	1.782	0.788	4.069	1.171
48	3.118	0.849	3.716	0.716	3.948	0.679	2.655	0.998	2.810	1.561
49	3.240	1.019	3.393	0.718	3.631	0.841	1.750	0.639	3.268	1.236
50	2.689	0.974	2.973	0.951	3.256	0.853	2.321	0.411	2.786	1.182
Total	3.029	0.921	3.762	0.759	3.582	0.888	2.023	0.872	2.806	1.383

**Table 7:** Apps included in the analyses

ID	App name	App in PlayGoogle	Download Date	Users rate	Comments number	Installations	In-app purchase	Developer website	Last Updated	Content category
1	Numbers for kids - learn to count 123 games!	https://play.google.com/store/apps/details?id=com.gokids.livenumbers	25/2/2021	4,1	4839	5.000.000 +	€1.59 - €9.99 per item	yes	November 3, 2020	Mathematics
2	Preschool learning games for toddlers & kids	https://play.google.com/store/apps/details?id=com.gokids.academy	27/2/2021	4,1	1,887	500.000+	€2.29 - €46.99 per item	yes	April 9, 2020	Mixed
3	Learning numbers for kids - kids number games!	https://play.google.com/store/apps/details?id=com.oki.numbers	27/2/2021	4	20,778	5.000.000+	€0.79 - €4.99 per item	yes	October 5, 2020	Mathematics
4	Learn Shapes for Kids, Toddlers - Educational Game	https://play.google.com/store/apps/details?id=com.oki.shapesnew	27/2/2021	4,2	7,553	1.000.000+	€0.79 - €4.99 per item	yes	March 4, 2020	Mathematics
5	Learning Transport Vehicles for Kids and Toddlers	https://play.google.com/store/apps/details?id=com.gokids.transport	27/2/2021	4,1	1,037	500.000+	-	yes	December 8, 2020	Mathematics
6	Learning Colors - Interactive Educational Game	https://play.google.com/store/apps/details?id=com.gokids.colorsrevenge	27/2/2021	4,3	322	100.000+	€0,99 - €4,99 per item	yes	May 18, 2020	Language
7	Shapes for Children - Learning Game for Toddlers	https://play.google.com/store/apps/details?id=com.oki.shapes	27/2/2021	3,8	12,615	1.000.000+	€0,79 - €5,30 per item	yes	August 26, 2019	Mathematics
8	Colors and Shapes for Toddlers	https://play.google.com/store/apps/details?id=comm.apps.pic4kids	27/2/2021	4,3	1,159	500.000+	€0,59 per item	yes	September 4, 2020	Mixed
9	Pocket Worlds - Learning Game	https://play.google.com/store/apps/details?id=air.com.tuxedogames.kidsgame	27/2/2021	4,4	270	50.000+	€1,99 - €7,64 per item	yes	March 16, 2021	Language
10	Toddler Preschool Educational Baby Games for Kids	https://play.google.com/store/apps/details?id=com.cubicfrog.toddlers.kindergarten.school	27/2/2021	4,2	185	50.000+	€10,99 - €54,99 per item	yes	February 23, 2020	Mixed
11	123 Basic Number Counting Math Games-EduMath1 Kids	https://play.google.com/store/apps/details?id=com.cubicfrog.edumath1	10/3/2021	4,2	127	10.000+	-	yes	June 5, 2017	Mathematics
12	Kids Counting Games: Kids 123 Counting Goobee	https://play.google.com/store/apps/details?id=com.goobee.GooBeeNumberGame	10/3/2021	4,1	461	100.000+	1.00€ per item	yes	March 18, 2018	Mathematics
13	Kids Preschool Learning Numbers & Maths Games	https://play.google.com/store/apps/details?id=com.greysprings.preschoolnumbers	10/3/2021	4,0	1000	100.000+	1,34€ per item	yes	July 14, 2014	Mathematics
14	123 Numbers Activity for Children | Kids Counting	https://play.google.com/store/apps/details?id=com.kindergarteneducationist.androidknb	10/3/2021	4,4	25	10.000+	0.89€ − 4.09€ per item	yes	September 8, 2018	Mathematics
15	Kids Kindergarten Math Lite	https://play.google.com/store/apps/details?id=com.intellijoy.kids.math.lite	10/3/2021	4,2	34	5000+	-	yes	February 10, 2021	Mathematics
16	Kids Learn Colors Lite	https://play.google.com/store/apps/details?id=com.anahoret.android.colors.lite	10/3/2021	3,9	41	50.000+	-	yes	December 3, 2020	Mixed
17	Colors & Shapes Game - Fun Learning Games for Kids	https://play.google.com/store/apps/details?id=com.greysprings.shapesandcolors	10/3/2021	4,1	764	100.000+	1.35€ per item	yes	April 7, 2021	Mixed
18	Dot to dot Game - Connect the dots ABC Kids Games	https://play.google.com/store/apps/details?id=com.greysprings.connect.dot.to.dot.ABC.games.kids	10/3/2021	3,7	224	100.000+	€ 1,49 per item	yes	February 19, 2021	Mixed
19	Animal Math Kindergarten Math Games for Kids Free	https://play.google.com/store/apps/details?id=com.eggrollgames.animalmathkindergartenfree	10/3/2021	4,1	55	10.000+	€4,59 per item	yes	May 4, 2020	Mathematics
20	Kindergarten and Preschool Montessori Kids Games	https://play.google.com/store/apps/details?id=com.queleas.preschoolgamesforkids2	10/3/2021	3,9	154	50.000+	€1,09 − 2,19 per item	yes	January 14, 2020	Language
21	Kindergarten Learning School	https://play.google.com/store/apps/details?id=com.sqsapps.kindergartenlearningschool	10/3/2021	4,0	122	100.000+	-	yes	November 9, 2020	Mixed
22	Kids Math - Kindergarten	https://play.google.com/store/apps/details?id=com.ramkystech.android.kidsmathkindergarten	10/3/2021	4,1	598	100.000+	-	yes	September 1, 2016	Mathematics
23	Kindergarten Maths - Count, add, subtract to 30	https://play.google.com/store/apps/details?id=au.com.parrotfish.math1	10/3/2021	3,8	18	10.000+	0,69€ per item	yes	March 21, 2021	Mathematics
24	Phonics - Sounds to Words for beginning readers	https://play.google.com/store/apps/details?id=au.com.parrotfish.phonemic.lite	10/3/2021	4,2	356	100.000+	0,69€ per item	yes	March 17, 2021	Language
25	Kids Learning Box: Preschool	https://play.google.com/store/apps/details?id=com.kidsappbox.kidslearninggame	10/3/2021	4,4	3,917	1.000.000+	3,19€ per item	yes	June 24, 2020	Mixed
26	Kids Learn - Quick ABC Learning | English Reading	https://play.google.com/store/apps/details?id=com.dts.kidsapp	10/3/2021	4,8	68	5.000+	-	yes	January 27, 2021	Language
27	First Grade Math (Lite)	https://play.google.com/store/apps/details?id=com.intellijoy.math.firstgrade.wp.lite	10/3/2021	4,1	644	100.000+	-	yes	December 14, 2020	Mathematics
28	Kindergarten Learning Games	https://play.google.com/store/apps/details?id=com.thelearningapps.kindergartenactivities	10/3/2021	4,3	115	50.000+	3.43€ per item	yes	April 8, 2021	Mixed
29	Kids Learn to Sort Lite	https://play.google.com/store/apps/details?id=com.intellijoy.sorting.lite	10/3/2021	3,8	340	100.000+	-	yes	December 9, 2020	Mixed
30	Kids ABC and Counting	https://play.google.com/store/apps/details?id=au.com.espace.dots.alphaNum	10/3/2021	4,2	5.000+	1.000.000+	2.29€ per item	yes	September 21, 2016	Mixed
31	Bedtime Math	https://play.google.com/store/apps/details?id=com.twofours.bedtimemath	23/3/2021	3,8	363	100.000+	-	yes	July 20, 2019	Mathematics
32	MiniMath by Bedtime Math	https://play.google.com/store/apps/details?id=org.BedtimeMath.Minimath	23/3/2021	0	0	1000+	-	yes	April 20, 2019	Mixed
33	Khan Academy Kids: Free educational games & books	https://play.google.com/store/apps/details?id=org.khankids.android&hl=el&gl=US	1/4/2021	4,5	21,000	1.000.000+	-	yes	February 5, 2021	Mixed
34	Sorting puzzles 2: Pre-k preschool learning games	https://play.google.com/store/apps/details?id=mobi.ka.th.sorting2.free	1/4/2021	4,2	29	10.000+	3,19 € per item	yes	April 28, 2020	Mixed
35	Little Panda Travel Safety	https://play.google.com/store/apps/details?id=com.sinyee.babybus.travelsafety&hl=el&gl=US	1/4/2021	4,1	37,425	10.000.000+	€ 0,50 per item	yes	January 28, 2021	Mixed
36	Kids Educational Puzzles Free (Preschool)	https://play.google.com/store/apps/details?id=com.forqan.tech.OwisPuzzles.free	1/4/2021	3,9	2,882	1.000.000+	2,89 per item	yes	October 12, 2020	Mixed
37	Not Like the Others Kids Game	https://play.google.com/store/apps/details?id=com.hedgehogacademy.notliketheotherslite	1/4/2021	4,5	4,146	1.000.000+	3,29 per item	yes	March 23, 2021	Language
38	Kids Spelling & Reading Games - Learn To Read	https://play.google.com/store/apps/details?id=com.iz.kids.word.search.games.spelling.words.vocabulary.letters.kindergarten.preschool.learning&hl=el&gl=US	1/4/2021	4,7	46	5.000+	10,99 per item	yes	February 1, 2021	Language
39	ParrotFish - Sight Words Reading Games	https://play.google.com/store/apps/details?id=au.com.sightwords.parrotfish.lite&hl=el&gl=US	1/4/2021	4,1	371	100.000+	0.69 per item	yes	March 21, 2021	Language
40	TinyTap - Educational Games for Kids, by Teachers.	https://play.google.com/store/apps/details?id=tinytap.kids.learning.games	1/4/2021	4,1	5,746	1.000.000+	10.99–124.99 per item	yes	March 15, 2021	Mixed
41	Preschool Adventures-3	https://play.google.com/store/apps/details?id=com.forqan.tech.families3	1/4/2021	4,0	1,392	500.000+	2.39 per item	yes	July 18, 2020	Mixed
42	Kids Math - Math Game for Kids	https://play.google.com/store/apps/details?id=com.dado.Addition.MathGameForKids&hl=el&gl=US	1/4/2021	4,1	3,841	1.000.000+	3.49 per item	yes	September 11, 2019	Mathematics
43	Learn ABC Alphabets	https://play.google.com/store/apps/details?id=vmobify.abc_alphabet_learning_game	1/4/2021	4,6	459	50.000+	0,99 per item	yes	June 27, 2020	Mixed
44	Preschool games for little kids	https://play.google.com/store/apps/details?id=com.bimiboo.adventure	1/4/2021	4,3	8,38	1.000.000+	3,99 per item	yes	January 29, 2020	Mixed
45	ABC Games for Kids – ABC Phonics	https://play.google.com/store/apps/details?id=com.idz.learnenglish	1/4/2021	4,2	73	10.000+	5,94 − 39,99 per item	yes	November 11, 2020	Language
46	Preschool Learning Games for Kids & Toddlers	https://play.google.com/store/apps/details?id=com.greysprings.games	1/4/2021	4,2	9,295	1.000.000+	2,39 per item	yes	April 5, 2020	Mixed
47	Kindergarten: animals	https://play.google.com/store/apps/details?id=com.YovoGames.Kindergarten	1/4/2021	4,1	16,072	5.000.00+	4,99€ per item	yes	August 19, 2020	Language
48	Kindergarten kids Learn Rhyming & Sight Word Games	https://play.google.com/store/apps/details?id=com.greysprings.wordgames	1/4/2021	3,9	413	100.000+	1,87€ per item	yes	April 8, 2021	Language
49	Kids Educational Games: Preschool and Kindergarten	https://play.google.com/store/apps/details?id=air.com.shubi.LearnCNLW.english	1/4/2021	4,3	4,394	1.000.000+	2,69€ per item	yes	January 18, 2020	Mixed
50	Kindergarten School Teacher	https://play.google.com/store/apps/details?id=com.minigamersclub.kindergartenschoolteacher	1/4/2021	4,2	632	100.000+	-	yes	July 26, 2020	Mixed
